# Feasibility and reliability of the pandemic-adapted online-onsite hybrid graduation OSCE in Japan

**DOI:** 10.1007/s10459-023-10290-3

**Published:** 2023-10-18

**Authors:** Satoshi Hara, Kunio Ohta, Daisuke Aono, Toshikatsu Tamai, Makoto Kurachi, Kimikazu Sugimori, Hiroshi Mihara, Hiroshi Ichimura, Yasuhiko Yamamoto, Hideki Nomura

**Affiliations:** 1 Medical Education Research Center, Kanazawa University, Kanazawa, Japan; 2 Department of Molecular Genetics, Kanazawa University, Kanazawa, Japan; 3 Department of Viral Infection and International Health, Kanazawa University, Kanazawa, Japan; 4 Department of Biochemistry and Molecular Vascular Biology, Faculty of Medicine, Institute of Medical, Pharmaceutical, and Health Sciences, Kanazawa University, Kanazawa, Japan; 5 Center for the Advancement of Higher Education, Hokuriku University, Kanazawa, Japan; 6 Center for Medical Education and Career Development, Toyama University, Toyama, Japan; 7 Department of General Medicine, Kanazawa University Hospital, 13-1 Takara-machi, Kanazawa, Ishikawa 920-8641 Japan

**Keywords:** Online objective structured clinical examination (OSCE), Reliability, Generalizability theory, Pandemic, Audiovisual conferencing system

## Abstract

Objective structured clinical examination (OSCE) is widely used to assess medical students’ clinical skills. Virtual OSCEs were used in place of in-person OSCEs during the COVID-19 pandemic; however, their reliability is yet to be robustly analyzed. By applying generalizability (G) theory, this study aimed to evaluate the reliability of a hybrid OSCE, which admixed in-person and online methods, and gain insights into improving OSCEs’ reliability. During the 2020–2021 hybrid OSCEs, one examinee, one rater, and a vinyl mannequin for physical examination participated onsite, and a standardized simulated patient (SP) for medical interviewing and another rater joined online in one virtual breakout room on an audiovisual conferencing system. G-coefficients and 95% confidence intervals of the borderline score, namely border zone (BZ), under the standard 6-station, 2-rater, and 6-item setting were calculated. G-coefficients of in-person (2017–2019) and hybrid OSCEs (2020–2021) under the standard setting were estimated to be 0.624, 0.770, 0.782, 0.759, and 0.823, respectively. The BZ scores were estimated to be 2.43–3.57, 2.55–3.45, 2.59–3.41, 2.59–3.41, and 2.51–3.49, respectively, in the score range from 1 to 6. Although hybrid OSCEs showed reliability comparable to in-person OSCEs, they need further improvement as a very high-stakes examination. In addition to increasing clinical vignettes, having more proficient online/on-demand raters and/or online SPs for medical interviews could improve the reliability of OSCEs. Reliability can also be ensured through supplementary examination and by increasing the number of online raters for a small number of students within the BZs.

## Introduction

Objective structured clinical examination (OSCE) is a clinical assessment tool used worldwide to evaluate undergraduate and postgraduate healthcare professionals (Patrício et al., 2013; Rentschler et al., 2007; Gormley, 2011). It is a test that can evaluate the clinical skills of medical students directly; however, there is a trade-off between its reliability and feasibility, as the former depends on the number of assessors, standard patients (SPs), clinical scenarios, and items that can be prepared on the same day at the same location.

Generalization (reliability) has been included in the four essential components of Kane’s (2006) validity argument (Cook et al., 2015). Although Cronbach’s alpha, the indicator of internal consistency (reliability), was considered as the primary basis for launching the USMLE Step 2 CS in 2004 (Papadakis, 2004), it could not comprehensively represent the error included in the raw scores of the examinees. In this regard, generalizability (G) theory makes it possible for researchers to assess the magnitude of error arising from each facet simultaneously, identify the greatest source of error, and optimize overall reproducibility by exploring the impact of varying numbers of replication for each facet (Brennan, 2001; Cook et al., 2015). The overall G- or Phi-coefficients of the G-theory estimate the reliability of the test and effects of variable errors by facets (Brennan, 2001; Tavakol & Brennan, 2013; Monteiro et al., 2019). The G-coefficient is an indicator used in examinations to determine rankings, and the Phi-coefficient is the score itself. G- or Phi-coefficient is described between 0 and 1, and the test is considered as having high reliability when the G- or Phi-coefficient is close to 1. Although G- or Phi-coefficient differs according to the purpose of the test, a G- or Phi-coefficient of 0.90 or higher (which indicates that the examinee’s performance was reflected on $\ge$90% of the total variance) is generally desirable for very high-stakes examinations such as the licensure examination because the test result has a significant consequence on the examinees and society (Downing, 2004). G-coefficient has been considered appropriate for use in pass/fail examinations when a summative OSCE is an examination that determines whether a student is above or below an imaginary student who is just on the borderline (Brennan, 2001; Cronbach & Shavelson, 2004; Bloch & Norman, 2012). Previously reported G-coefficients of summative OSCEs were relatively low, ranging from 0.50 to 0.84 (Baig & Violato, 2012; Vallevand & Violato, 2012; Park et al., 2016), except for one (G-coefficient 0.93) of an 18-station OSCE (Trejo-Mejía et al., 2016). Increasing the number of stations and raters could help improve the reliability of OSCEs (Brannick et al., 2011); however, this often jeopardizes the feasibility of the OSCE itself (Moss et al., 1994). Thus, it is important to increase the reliability of OSCE without a decrease in feasibility.

Unintentionally, the feasibility and validity of the OSCE are once again under debate in the era of the COVID-19 pandemic (Malau-Aduli et al., 2022). The COVID-19 pandemic has complicated the administration of face-to-face OSCEs that involve dozens of SPs and raters. Many educational facilities for healthcare professionals, including residents, medical, nursing, pharmacy, and dentistry students, rapidly transitioned from traditional in-person OSCEs to virtual OSCEs (also described as remote OSCE, online OSCE, or teleOSCE) (Felthun et al., 2021a; Malau-Aduli et al., 2022). These virtual OSCEs are satisfactory, feasible, and cost-effective as per survey results (Langenau et al., 2014; Shaban et al., 2021; Shaiba et al., 2021), questionnaire feedbacks (Skrzypek et al., 2022; Motkur et al., 2022; Garofalo et al., 2023), empirical analysis (Felthun et al., 2021b; Minty et al., 2022), and descriptive qualitative studies (Shorbagi et al., 2022; Saad et al., 2022; Thampy et al., 2022;). Although three studies assessed the reliability of virtual OSCEs by calculating Cronbach’s alphas, and they were about 0.80 (Oliven et al., 2011, 2021; Porto et al., 2023), a reliability analysis based on G-theory is yet to be reported (Kunutsor et al., 2022).

This study aims to evaluate the reliability of summative OSCE using the onsite-online hybrid system by applying G-theory and to show that OSCEs can be conducted during a pandemic without losing reliability. Based on the analysis by G-study, the study also clarified the facets or the interactions among facets that should be promptly dealt with to improve the reliability of OSCEs. In addition, the study conducted a decision (D) study to search for an optimal setting of OSCE to achieve G-coefficients of 0.90 or higher as a very high-stakes examination in the hybrid setting. The data in the present research could be useful to implement hybrid OSCE with high reliability and feasibility as well as to obtain insights to improve the reliability of conventional in-person OSCEs, which were not sufficient in the first place.

## Methods

### Design

This study is a retrospective analysis of a pre-existing dataset obtained from the five-year post-clinical clerkship (post-CC) OSCEs at a single medical school in Japan, before (2017–2019) and during (2020–2021) the COVID-19 pandemic. The Research Ethics Committee of Kanazawa University approved the present study (No. 2764).

### Subjects and setting

During 2017–2021, all medical students in their final (sixth) year at Kanazawa University School of Medicine (n = 110–124) were divided into 8–11 circuits of 3–6 stations, where different case vignettes were applied (Table 1). In Japan, the post-CC OSCE with six stations, organized by the Common Achievement Test Organization (CATO) and administered by each medical school, was expected to be implemented in all medical schools in 2020 after a three-year trial from 2017 to 2019 as an academic requirement for graduation. Thus, the number of stations had gradually increased during OSCE trials, and the number of raters had also increased accordingly. Regarding the number of stations in 2020 and 2021, the implementation of the hybrid OSCE required the preparation of more rooms and IT equipment connected to a secured local area network and a modified schedule to avoid problems with the IT equipment; hence, three stations were the limit.

**Table 1:** Settings of post-CC OSCE in each year

	2017	2018	2019	2020	2021
Student (p)	121	110	124	110	116
Circuit (c)	10	11	11	8	8
Station (S)	3	4	6	3	3
Rater (R)	1.5	2	2	2	2
Item (I)	6	6	6	6	6

**Fig. 1:**
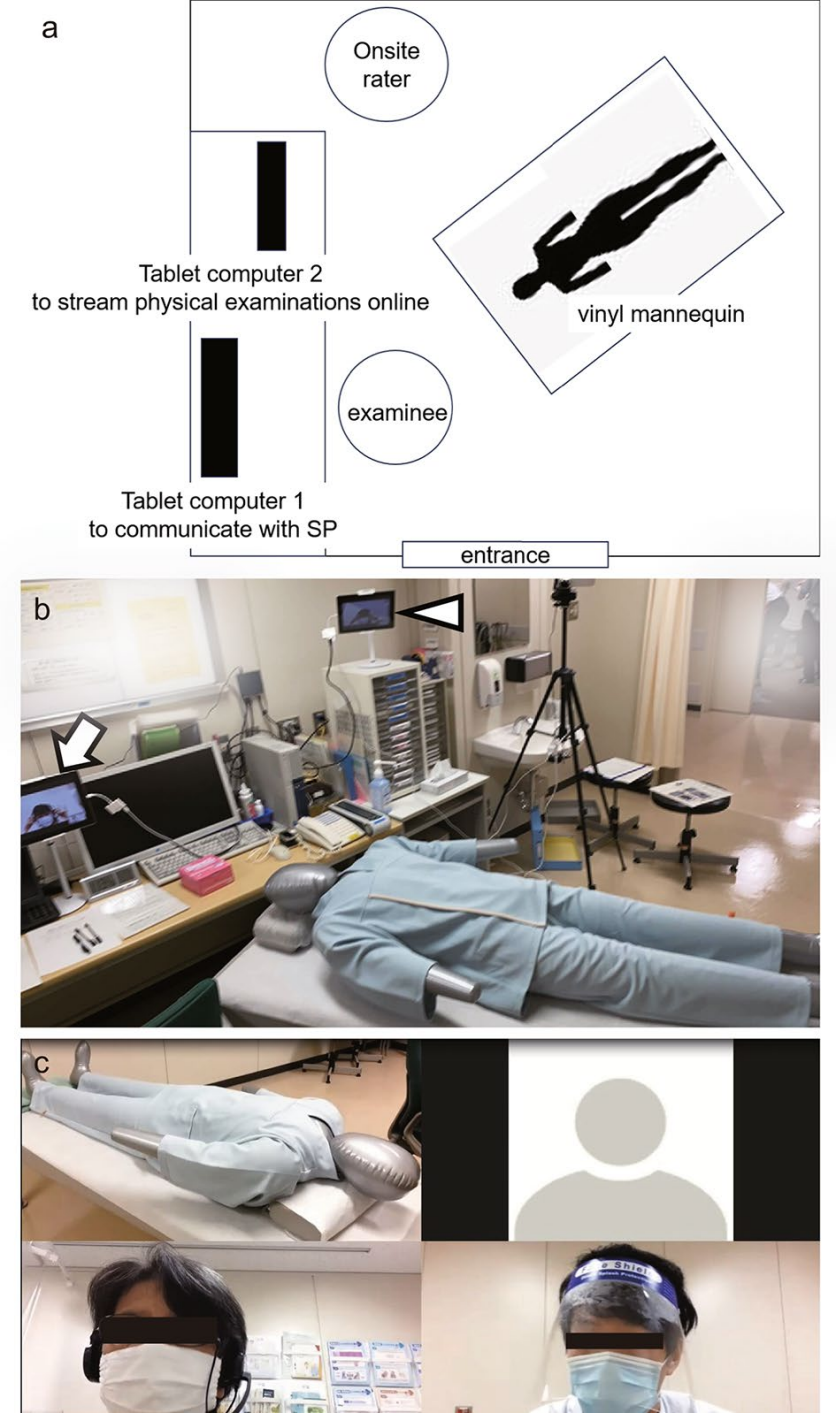
The setting of post-CC OSCE in 2020 and 2021. **a** and **b**: Setting in a test room. The examinee sitting at the desk can communicate with the standardized patient displayed on a tablet computer (tablet computer 1; arrow). A vinyl mannequin lies on the examination table, and physical examination performance is real-time streamed to the online rater through the other tablet computer (tablet computer 2; arrowhead). **c**: A screenshot of the laptop computer display for the online rater. The student (lower right) and the standardized patient (lower left) hold a conversation during history taking. Then, the online raters watch the student performing a physical examination with a vinyl mannequin (upper left). Finally, the raters, one in the test room and the other in a separate room, listen to and assess the case presentation of the student

In conventional in-person OSCEs conducted during 2017–2019, each test room was assigned to the examinee, one SP for the medical interview, another SP for physical examination, and two raters. In pandemic-resistant hybrid OSCEs conducted during 2020–2021, each test room was assigned to the examinee, one of the two raters, and a vinyl mannequin for physical examination. Two other separate rooms were networked as one virtual breakout room on a web-based audiovisual conferencing system (Zoom Video Communications, San Jose, CA). SP for the medical interview was in the second room, and the other rater in the third. The test room was equipped with two tablet computers (Apple, Los Altos, CA), one for conducting online medical interviews and the other for streaming the physical examination (Fig. 1a and b). The online rater observed the student’s performance on a laptop computer (Dell Technologies, Round Rock, TX) display and recorded it for future verification (Fig. 1c). All tablets and laptop computers were wired to the campus area network at Kanazawa University.

During 2017–2021, the tests implemented 3, 4, 6, 3, and 3 case vignettes, respectively. After listening to a short instruction, students were directed to read the basic case information, including age, gender, and reason for the encounter, for the first minute. Subsequently, the students called in the SPs and performed a medical interview and physical examination for 12 min. In 2017, after completing the physical examination, they spent four minutes preparing and another four minutes presenting the case, assessment, differential diagnoses, and management plan. During 2018–2021, after completing the physical examination, students spent four minutes preparing and presenting the case, assessment, and differential diagnoses.

### Raters and rating scale

A total of 60, 70, 87, 48, and 48 raters engaged in the post-CC OSCEs in 2017, 2018, 2019, 2020, and 2021, respectively. Sixty minutes of rater training was provided to all raters within one month before the examination. In the 2017 OSCE, 30 test rooms were divided into 10 circuits of three stations. In one of the three stations per circuit, one of the two raters used assessment sheet A (global rating scales), and the other used assessment sheet B (checklists). In the other two stations, both raters used assessment sheet A. Thus, the 10 raters who used assessment sheet B were excluded from the analysis. By excluding these 10 raters, the harmonic mean of the number of raters per test room was 1.5 in 2017.

On assessment sheet A, a 6-point global rating scale (1, poor and needs general re-education; 2, poor and needs partial re-education; 3, borderline; 4, pass and expected level at the start of postgraduate training; 5, good and expected level in the middle of postgraduate clinical training; and 6, excellent and above the level expected at the end of postgraduate training) was used for all six items (A, generosity and communication skills; B, history taking; C, physical examination based on diagnostic hypothesis; D, case presentation; E, clinical reasoning; F, holistic assessment). In subsequent years, all raters used assessment sheet A.

### Standardized simulated patients

A total of 50, 52, 68, 31, and 34 SPs for medical interviews participated in the examinations in 2017, 2018, 2019, 2020, and 2021, respectively. Experienced SPs had a prior training session of at least 90 min, and other SPs participated in 60-minute training sessions twice and a 30-minute training session immediately before the examination. The conductors confirmed that all SPs showed acceptable performance during training sessions.

During 2017–2019, SPs for physical examination were recruited from residents, fifth-year medical students, and students belonging to other non-healthcare schools at Kanazawa University. They received a 20-minute brief immediately before the examination because they only had to know how to behave in the examination room and move between the examination and waiting rooms.

### Statistical analysis

The possible raw scores of the examinees were calculated as the mean marks of each item (A, B, C, D, E, and F) for all stations; the average of mean marks of items A, B, C, D, and E (item A–E) for all stations; and the average of mean marks of items A, B, C, D, E, and F (item A–F) for all stations. An exploratory factor analysis was performed using the mean scores for each item in the OSCE 2021 data to determine if each item measured was related to the same single factor or if multiple factors were extracted (Watkins, 2018). The Kaiser-Meyer-Olkin measure was calculated to assess the sampling adequacy of this exploratory factor analysis. The Kaiser-Meyer-Olkin values ranged from 0.00 to 1.00, and overall Kaiser-Meyer-Olkin value ≥ 0.70 was considered appropriate (Watkins, 2018).

G-studies were carried out to determine the reliability of students’ raw scores in the examination and the magnitude of the identified sources of variance. A five facet, (p:c)⨯(R:S)⨯I design was adopted where p, c, R, S, and I denote students, circuits, raters, stations, and items on assessment sheets, respectively, when the average of mean marks for items A–E or that of mean marks for items A–F were used as the examinees’ raw scores. Students (p) were the facet of differentiation and stratified by circuits (p:c). Raters, stations, and items on the assessment sheets were the facets of generalization. Raters were nested within stations (R:S). Another model, (p:c)⨯(R:S), was adopted when analyzing the reliability of each assessment item (A–F). The variance components (VC) of each facet and the interaction of facets were computed using urGENOVA developed by Brennan (2001).

G-coefficient is the ratio of universe score variance to itself plus relative error variance (Brennan, 2001). G-coefficients were calculated manually, according to Brennan, as indicators for consistency. Phi-coefficient, which is the index of dependability, is the ratio of universe score variance to itself plus absolute error variance (Brennan, 2001). Phi-coefficients were also calculated to derive the standard error of measurements (SE_m_) by $Standard Deviation\times\sqrt{1-Phi}$. The estimated 95% confidence intervals (CIs) of the score of an imaginary borderline student, namely border zones (BZs), were calculated by mean ± 1.96⨯SE_m_.

D-studies were also conducted to examine the G-coefficients when the numbers of stations, raters, or items were changed virtually. They make it possible to compare G-coefficients in different settings of OSCEs. The study performed two D-studies. First, G-coefficients under a standardized setting of six stations, two raters, and six items determined by CATO were compared between in-person OSCEs (2017–2019) and hybrid OSCEs (2020 and 2021). Second, a D-study was performed in hybrid OSCE 2021 to determine what settings can achieve G-coefficients of 0.90 or higher.

The marks by the online and onsite raters in 2020 and 2021 were compared using the paired Wilcoxon rank-sum test. The significance level for the analyses was set at 0.05.

## Results

### Trends in G-coefficients during 2017–2021

First, an exploratory factor analysis with the mean marks of each item for all stations to validate the item using the data of hybrid OSCE in 2021 was performed. Prior to an exploratory factor analysis, the study calculated a Kaiser-Meyer-Olkin measure to assess the sampling adequacy, and it was 0.815, confirming enough sampling adequacy. Exploratory factor analysis showed that one factor was associated with all items measured (Table 2). In addition, the G-coefficients were calculated when the raw score of each examinee was calculated with each assessment item alone (score A, B, C, D, E, and F), with all items except for item F (score A–E), and with all items including item F (score A–F) (Table 2). As a result, score F, which reflects the item F score of the holistic assessment, showed the highest G-coefficient (0.747), whereas score A–F was the second highest (0.733). Although the study applied score A–F as the raw score of each examinee for further analyses, as determined in advance, score F could be an alternative as the raw score of each examinee of OSCE.

**Table 2:** The factor loadings for factor 1 and G-coefficients for each possible raw score in OSCE 2021

Raw score	Factor loadings for factor 1	G-coefficient
Score A	0.686	0.658
Score B	0.670	0.525
Score C	0.760	0.650
Score D	0.866	0.698
Score E	0.768	0.708
Score F	0.993	0.747
Score A–E		0.703
Score A–F		0.733

Abbreviations: Score A (B, C, D, E, and F), the raw score calculated with item A (B, C, D, E, and F, respectively) marks alone; Score A–E (A–F), the raw score calculated with item A, B, C, D, and E (A, B, C, D, E and F) marks

**Fig. 2:**
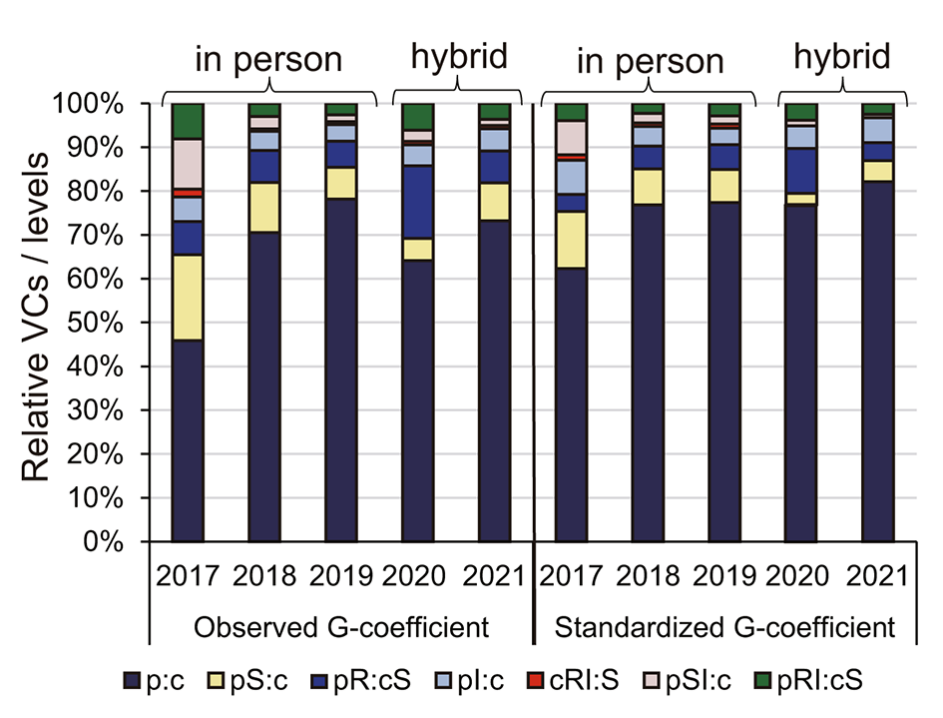
Trends in observed and standardized G-coefficients. G-coefficients in actual setting (observed G-coefficient) and those in standard setting of 6-station, 2-rater, and 6-item (standardized G-coefficient) were depicted in each year. In each bar chart, the relative proportions of variance components per level of each facet are drawn so that the stack is 100%. Thus, the fractions of [p:c] the bars edged with bold black lines show G-coefficients in percentage. Abbreviations: c, circuits; I, items; p, students; R, raters; S, stations; p:c, students stratified by circuits; VC, variance component : means stratified by or nested within ⨯ means crossed with

Subsequently, a G-study was performed to elucidate the validity of in-person and hybrid OSCEs. The VC of each facet or interaction of facets and their fraction to calculate G-coefficients during 2017–2021 are shown in Tables 3 and 4; Fig. 2. The fraction of [p:c] is the G-coefficient of the OSCE each year. The G-coefficients of the OSCEs observed were 0.459, 0.705, and 0.782, in in-person OSCEs of 2017, 2018, and 2019, respectively (Table 3; Fig. 2), while 0.641 and 0.733, in hybrid OSCEs of 2020 and 2021, respectively (Table 4; Fig. 2). Both in in-person and hybrid OSCEs, G-coefficients gradually increased year by year, although the values were still under 0.80. Among the source of variance, the interaction between student and station was the largest variable errors in 2017, 2018, 2019, and 2021 (19.6%, 11.5%, 7.3%, and 8.5%, respectively) (Tables 3 and 4), while interaction between student and rater was the largest in 2020 (16.6%) (Table 4). These results suggest that interaction between student and station and student and rater were the main factors that decreased the reliability of OSCEs.

**Table 3:** VC of each source of variance, and proportion of variance explained among the sum of VC/level in in-person post-CC OSCEs

Source of variance	2017					2018					2019				
	df	SS	MS	VC	VC/level (%)	df	SS	MS	VC	VC/level (%)	df	SS	MS	VC	VC/level (%)
[p:c]	111	340.3	3.066	0.048	45.9	100	699.5	6.995	0.103	70.5	113	844.1	7.470	0.082	78.2
[p:c]⨯S	222	302.5	1.363	0.061	19.6	300	519.5	1.732	0.067	11.5	565	737.7	1.306	0.046	7.3
[p:c]⨯[R:S]	222	98.0	0.441	0.035	7.6	400	289.5	0.724	0.086	7.3	678	433.8	0.640	0.074	5.9
[p:c]⨯I	555	436.4	0.786	0.035	5.6	500	358.5	0.717	0.038	4.3	565	337.8	0.598	0.024	3.8
c⨯[R:S]⨯I	90	74.9	0.832	0.050	1.8	180	109.0	0.605	0.036	0.5	300	224.8	0.749	0.049	0.7
[p:c]⨯S⨯I	1110	628.4	0.566	0.215	11.5	1500	617.3	0.412	0.101	2.9	2825	884.2	0.313	0.059	1.6
[p:c]⨯[R:S]⨯I	1075	237.6	0.221	0.226	8.1	2000	418.2	0.209	0.209	3.0	3390	660.5	0.195	0.195	2.6

Abbreviations: VC, variance component; df, degree of freedom; SS, sum of squares; MS, mean sum of squares; p, students; c, circuits; R, raters; S, stations; I, items; [p:c] means p stratified by c; [R:S] means R nested within C; ⨯ means crossed with

**Table 4:** VC of each source of variance, and proportion of variance explained among the sum of VC/level in hybrid post-CC OSCEs

Source of variance	2020					2021				
	df	SS	MS	VC	VC/level (%)	Df	SS	MS	VC	VC/level (%)
[p:c]	109	362.7	3.327	0.060	64.1	107	525.2	4.908	0.101	73.3
[p:c]⨯S	218	221.9	1.018	0.014	5.1	214	224.7	1.050	0.035	8.5
[p:c]⨯[R:S]	327	248.5	0.760	0.093	16.6	321	177.3	0.552	0.059	7.1
[p:c]⨯I	545	245.8	0.451	0.027	4.8	525	276.7	0.517	0.040	4.8
c⨯[R:S]⨯I	105	62.1	0.591	0.026	0.8	105	69.3	0.660	0.032	0.6
[p:c]⨯S⨯I	1090	316.8	0.291	0.043	2.6	1070	296.6	0.277	0.040	1.6
[p:c]⨯[R:S]⨯I	1635	333.8	0.204	0.204	6.1	1605	315.7	0.197	0.197	4.0

Abbreviations: VC, variance component; df, degree of freedom; SS, sum of squares; MS, mean sum of square; p, students; c, circuits; R, raters; S, stations; I, items; [p:c] means p stratified by c; [R:S] means R nested within C; ⨯ means crossed with

### Descriptive statistics of the post-CC OSCE scores each year

The study ensuingly analyzed how many students were in the BZs where low reliability of the test could affect largely in OSCEs each year. The mean raw score, standard deviation, and the SE_m_ of OSCEs each year are shown in Table 5. The BZs in 2017–2021 would be 2.37–3.63, 2.51–3.49, 2.59–3.41, 2.53–3.47, and 2.46–3.54, respectively. During 2017–2021, there were 2, 18, 3, 6, and 4 students, respectively, who scored within these intervals. These results suggest that the scores of examinees and the number of students in BZs were not different between in-person and hybrid OSCEs. These students in BZs should be reassessed by a higher reliability method.

**Table 5:** Descriptive statistics of the post-CC OSCE scores each year

	2017	2018	2019	2020	2021
Mean raw score	4.31	3.87	4.21	4.11	4.23
SD	0.36	0.38	0.34	0.32	0.41
SE_m_	0.32	0.25	0.21	0.24	0.27
BZ	2.37–3.63	2.51–3.49	2.59–3.41	2.53–3.47	2.46–3.54
Number (%) in BZ	2 (1.7%)	18 (16.4%)	3 (2.4%)	6 (5.5%)	4 (3.4%)

Abbreviations: SD, Standard deviation of students’ scores; SE_m_, Standard error of measurement; C.I., confidence interval; BZ, 95% C.I. of borderline score (border zone)

Next, the study compared ratings between onsite and online raters in hybrid OSCEs. In 2020, online raters marked the scores similarly to onsite raters (4.12 ± 0.37 vs. 4.10 ± 0.41; p = 0.95). Contrarily, online raters marked the scores significantly yet slightly lower than onsite raters in 2021 (4.21 ± 0.44 vs. 4.25 ± 0.43; p = 0.042).

### G-coefficients under the standard setting by D-studies and facets in need of significant improvement

G-study cannot compare the quality of stations and raters in OSCE each year directly because different test settings, such as the number of stations and raters, affected the G-coefficients largely. Thus, the study performed D-studies to assess the G-coefficients under the standard setting [S = 6, R = 2, I = 6] determined by CATO. The VC of each facet or interaction of facets and their fractions under the standard setting [S = 6, R = 2, I = 6] are shown in Table 6. The G-coefficients under the standard setting (standardized G-coefficients) were 0.624, 0.770, 0.782, 0.759, and 0.823 in 2017, 2018, 2019, 2020, and 2021, respectively (Table 6; Fig. 2), suggesting that the reliability of hybrid OSCEs did not decrease as compared to in-person OSCEs under the standard setting. Notably, fractions of VC/levels of complex interactions such as [p:c]⨯[S]⨯[I], [c]⨯[R:S]⨯[I], as well as [p:c]⨯[R:S]⨯[I], are relatively small under the standard setting, whereas those of two-facet interactions such as [p:c]⨯[I], [p:c]⨯[S], as well as [p:c]⨯[R:S], accounted for 4–6% of the sum of all VC/levels in the hybrid OSCE 2021 (Table 6). These findings suggest that interaction between students and items, students and stations, and students and raters are major sources of variances that should be dealt with to improve the reliability of hybrid OSCEs.

**Table 6:** D-study results with datasets 2017–2021 when a standard [S = 6, R = 2, I = 6] setting was applied

Source of variance	2017		2018		2019		2020		2021	
	VC	VC/level (%)	VC	VC/level (%)	VC	VC/level (%)	VC	VC/level (%)	VC	VC/level (%)
[p:c]	0.048	62.4	0.103	77.0	0.082	78.2	0.060	75.9	0.101	82.3
[p:c]⨯S	0.061	13.3	0.067	8.3	0.046	7.3	0.014	3.0	0.035	4.8
[p:c]⨯[R:S]	0.035	3.9	0.086	5.2	0.074	5.9	0.093	9.8	0.059	4.0
[p:c]⨯I	0.035	7.6	0.038	4.7	0.024	3.8	0.027	5.7	0.040	5.4
c⨯[R:S]⨯I	0.050	0.9	0.036	0.4	0.049	0.7	0.026	0.5	0.032	0.4
[p:c]⨯S⨯I	0.215	7.8	0.101	2.1	0.059	1.6	0.043	1.5	0.040	0.9
[p:c]⨯[R:S]⨯I	0.226	4.1	0.209	2.2	0.195	2.6	0.204	3.6	0.197	2.2

Abbreviations: p, students; c, circuits; R, raters; S, stations; I, items; [p:c] means p stratified by c; [R:S] means R nested within C; ⨯ means crossed with; VC, variance component

The BZs under the standard setting in the OSCE 2017–2021 were also estimated to be 2.43–3.57, 2.55–3.45, 2.59–3.41, 2.59–3.41, and 2.51–3.49, respectively. When score F was applied as the raw score of each examinee of OSCE, the BZs were 2.47–3.53, 2.63–3.37, 2.64–3.34, 2.66–3.34, and 2.62–3.38, respectively. These results suggest that score F could alternate score A–F to make fail/pass determinations of examinees.

**Fig. 3:**
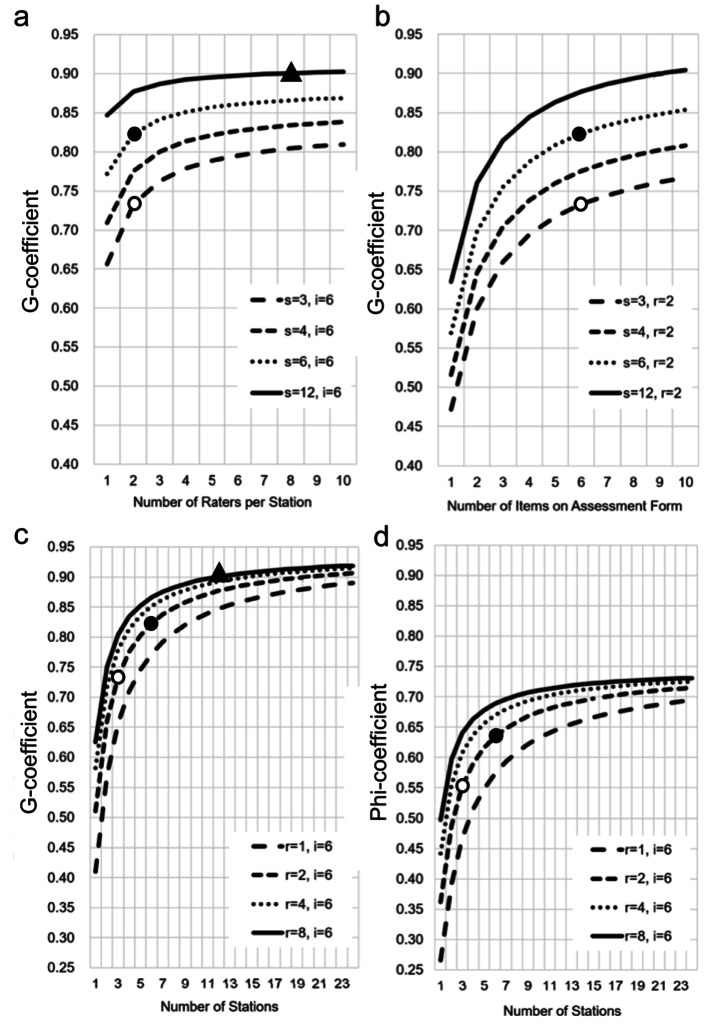
D-study results based on variance components derived from 2021 data. Effects of **(a)** the number of raters per station, **(b)** the number of items on the assessment form, and **(c)** the number of stations on G-coefficient are shown. Panel D shows the effect of the number of stations on Phi-coefficient. The observed G- and Phi-coefficients are marked with white circles (〇), where G- and Phi-coefficient under the simulated 6-station, 2-rater, 6-item setting are marked with black circles (●). The [S = 12, R = 8, I = 6] setting where a G-coefficient would exceed 0.90 is shown with black triangles (▲)

Finally, the study performed further D-study to elucidate the optimal setting to achieve a G-coefficient of $\ge$0.90 (Fig. 3). The results based on the VCs obtained in the analysis of the hybrid OSCE 2021 were used for this D-study because the hybrid OSCE 2021 showed the highest G-coefficient under standard setting (Table 6; Fig. 2). To achieve a G-coefficient larger than or equal to 0.90, a [S = 12, R = 8, I = 6] OSCE would be needed (Fig. 3). If score F was used as the raw score of each examinee, an [S = 6, R = 5, I = 1] or [S = 12, R = 2, I = 1] OSCE would be sufficient.

## Discussion and conclusions

To the best of our knowledge, this is the first study to report the generalizability (reliability) of onsite-online hybrid OSCEs by G-theory. After the announcement of the USMLE Step 2 CS discontinuation, the National Board of Medical Examiners admitted that they had considered developing a model for remote administration, but there were “challenges associated with technology, security, equity, and exam logistics, as well as the obvious limitations to assessing physical examination skills via a virtual performance assessment” (Katsufrakis & Chaudhry, 2021). Introducing new technologies requires evidence for both reliability and validity (Tsichlis et al., 2021). Furthermore, although post-CC OSCEs have recently returned to in-person style as the COVID-19 pandemic has been alleviated, the reliability of virtual OSCE should be estimated for the following reasons. First, we should prepare for future outbreaks of infectious diseases which may put us in similar situations as the COVID-19 pandemic. Second, in-person OSCE needs a large number of human resources, such as raters and SPs. In this regard, hybrid OSCEs could resolve the burden of increasing the number of stations and confirm the feasibility without reducing reliability. Third, outcomes of summative OSCE, such as post-CC OSCE in Japan are significant for both students and society because scores in post-CC OSCE determine the graduation of medical students. Therefore, the high reliability of the test is imperative for it to be trusted by society, even in an emergency like the COVID-19 pandemic. Additionally, since the reliability of post-CC OSCE in Japan is yet to be analyzed and published, the present study could help improve the reliability of post-CC OSCE in Japan.

The most notable point of this study is that the G-coefficients under the standard setting [S = 6, R = 2, I = 6] of hybrid OSCEs (2020–2021) were at least comparable to those of in-person OSCEs (Table 5; Fig. 2). Three studies have assessed the reliability of virtual OSCEs by calculating Cronbach’s alphas (Oliven et al., 2011, 2021; Porto et al., 2023). Porto et al. (2023) conducted virtual OSCE to assess oral radiology skills and competencies of undergraduate dentistry students, and its reliability was compared to that of in-person OSCE for the same participants. The Cronbach’s alphas of virtual OSCE was 0.81, while that of in-person OSCE was 0.61, showing substantial reliability. Oliven et al. (2011) have developed and assessed the utility of internet-based OSCE using virtual patients for medical students prior to the COVID-19 pandemic. The Cronbach’s alphas of the virtual patients’ OSCE were 0.82–0.89, while those of conventional human OSCE were 0.65–0.74 in 2008–2010, showing that the virtual patients’ OSCE had substantially higher reliability than conventional human OSCE. The authors also reported during the COVID-19 pandemic that Cronbach’s alphas of the virtual patients OSCE were 0.86 on average during 2015–2019 (Oliven et al., 2021). These data suggest that virtual OSCEs could be utilized as valid and reliable examination tools. Since Cronbach’s alpha is not fully sufficient to estimate the reliability as it reflects only internal consistency, the results of our analysis using G-theory could strengthen the evidence that virtual OSCEs are considered an adequate alternative to in-person OSCEs because G-theory reflects all sources of variance and those interactions in addition to internal consistency.

Applying G- and D-studies, the study clarified the major source of variances that decrease the reliability of hybrid OSCEs. Since G-coefficient in our hybrid OSCEs still did not reach the reliability criteria for a very high-stakes examination (G-coefficient ≥ 0.90) (Downing, 2004), we should deal with the obstacles to improve the reliability of OSCEs. The study found the three main obstacles; [p:c]⨯[I], [p:c]⨯[S], and [p:c]⨯[R:S] interactions (Tables 3 and 4, and 6). Although increasing the number of stations and raters would be a simple mathematical solution, doubling the stations would simply require doubling the raters and SPs as well as test rooms or days, leading to a decrease in feasibility. Thus, we need to consider other solutions to increase reliability while maintaining feasibility.

First, although our D-study showed that increasing the number of items improves G-coefficient logically (Fig. 3b), G-coefficient was surprisingly the highest when the study applied only score F, which is the score of the holistic assessment (item F), as the raw score of each examinee in 2021 hybrid OSCE (Table 2). Additionally, the study adopted the raw score calculated with all item marks (score A–F) since it had considered that item F alone might have less explanatory adequacy but it might add gestalt by the rater not included in score A–E; however, score F as the raw score could be an obvious alternative choice, considering the data in the present study. Nonetheless, there could be significant challenge to adopt only score F as the raw score of each examinee in terms of accountability to students.

Second, it is important to deal with the interaction between students and stations. Increasing the number of stations up to 12 would lead to the improvement of reliability with a G-coefficient of 0.90 or greater (Fig. 3). Since 12-station hybrid OSCE could lead to a huge burden for SPs, raters, and test rooms, providing supplementary examination for not all students but only students who fell within BZ could be a feasible alternative. The improvement of SP’s performance, as well as the quality of clinical vignettes, would reduce the variance error related to the station (Streiner et al., 2015). The fact that our SPs participate in post-CC OSCE activities once a year or less makes it difficult for them to maintain their performance at the highest level. In addition, any prior relationship between examinees and SPs could affect the variance of [p:c]⨯[S] interactions. Building a small pool of well-trained, highly experienced SPs who have no acquaintance with medical students except for OSCEs could be an ideal solution.

Last, increasing the number and quality of raters could improve the reliability of hybrid OSCEs. Regarding the number of raters, increasing up to eight raters could help achieve a 0.90 or higher G-coefficient in hybrid OSCE (Fig. 3a). Online OSCE has an advantage in providing readily accessible video recordings for additional raters. Our results showed that assessments of online raters were not largely different from those of onsite raters, although online raters scored a little lower than onsite raters in 2021 hybrid OSCE, supporting previous data (Chan et al., 2014; Chen et al., 2019; Bouzid et al., 2022). Thus, the assessment of additional raters using recorded videos for only students who fell within BZ could be a reasonable solution without reducing feasibility. In terms of the quality of the performance by raters, similar problems and solutions like the SPs have existed in raters as well. Our raters participate in post-CC OSCE once a year or less, and any prior relationship between examinees and rater could affect the variance of [p:c]⨯[R:S] interactions. Building a small pool of well-trained, highly experienced raters who have no acquaintance with medical students except for OSCEs could be an ideal solution. Setting the shared passing standard through an official consensus-making process should also be considered to improve the quality of ratings (Van der Vleuten & Cohen-Schotanus, 2009).

This study has some limitations. First, since students were stratified in 8 to 11 circuits, the G-coefficient provides the rank ordering of students limited to those in the same circuit. Second, as the present data are from a single medical school, the effect of variance among schools could not be assessed. Most circuits included actual medical examination rooms, which might have advantages in realism but disadvantages in standardization. Third, the marks in the physical examination assessment may not fully reflect the students’ true skills because we used vinyl mannequins without the most elaborate body parts in hybrid OSCEs to reduce the costs. Utilizing more elaborate simulators would be appropriate for physical examination assessment. Last, since D-studies are mathematical simulations, confirmation of the actual operation is required.

In conclusion, the generalizability of our online-onsite hybrid graduation OSCE is not inferior to the conventional in-person OSCE. Although it was still insufficient as a very high-stakes OSCE, hybrid OSCE could be an alternative to in-person OSCE. Efforts should be taken to diminish the variance in interactions between students and raters, students and stations, and students and assessment items. This issue could be addressed by inviting experienced raters to participate in remote online or video-on-demand assessment activities and by inviting well-trained SPs to participate in remote medical interviewing. Since the post-CC OSCE is a pass/fail exam, it would be possible to ensure the necessary reliability while avoiding a huge burden by taking measures to increase reliability, such as supplementary examinations and increasing the number of online raters for a small number of students within the BZs.

## References

[CR1] Baig LA, Violato C (2012). Temporal stability of objective structured clinical exams: A longitudinal study employing item response theory. BMC Medical Education.

[CR2] Bloch R, Norman G (2012). Generalizability theory for the perplexed: A practical introduction and guide: AMEE Guide No. 68. Medical Teacher.

[CR3] Bouzid, D., Mullaert, J., Ghazali, A., Ferré, V. M., Mentré, F., Lemogne, C., Ruszniewsk, P., Faye, A., Dinh, A. T., & Mirault, T. (2022). eOSCE stations live versus remote evaluation and scores variability. *BMC Medical Education*, *22*(1), 861. 10.1186/s12909-022-03919-1. Université Paris Cité Osce study group. 10.1186/s12909-022-03919-1 PMC9745699 36514011

[CR5] Brannick MT, Erol-Korkmaz HT, Prewett M (2011). A systematic review of the reliability of objective structured clinical examination scores. Medical Education.

[CR4] Brennan, R. L. (2001). *Generalizability theory*. Springer.

[CR6] Chan J, Humphrey-Murto S, Pugh DM, Su C, Wood T (2014). The objective structured clinical examination: Can physician-examiners participate from a distance?. Medical Education.

[CR7] Chen T, Lin M, Chiang Y, Monrouxe L, Chien S (2019). Remote and onsite scoring of OSCEs using generalizability theory: A three-year cohort study. Medical Teacher.

[CR8] Cook DA, Brydges R, Ginsburg S, Hatala R (2015). A contemporary approach to validity arguments: A practical guide to Kane’s framework. Medical Education.

[CR9] Cronbach LJ, Shavelson RJ (2004). My current thoughts on coefficient alpha and successor procedures. Educational and Psychological Measurement.

[CR10] Downing SM (2004). Reliability: On the reproducibility of assessment data. Medical Education.

[CR11] Felthun JZ, Taylor S, Shulrug B (2021). Assessment methods and the validity and reliability of measurement tools in online objective structured clinical examinations: A systematic scoping review. Journal of Educational Evaluation for Health Professions.

[CR12] Felthun JZ, Taylor S, Shulruf B, Allen DW (2021). Empirical analysis comparing the tele-objective structured clinical examination (teleOSCE) and the in-person assessment in Australia. Journal of Educational Evaluation for Health Professions.

[CR13] Garofalo LE, Gaikwad N, Menzies S, Thomas C, Curtis C, Parpal H (2023). prepOSCE: A virtual, scalable solution to prepare residents for their OSCE examination. Canadian Journal of Neurological Sciences.

[CR14] Gormley G (2011). Summative OSCEs in undergraduate medical education. The Ulster Medical Journal.

[CR15] Kane, M. T. (2006). Validation. In R. B. Brennan (Ed.), *Educational measurement* (4th ed., pp. 17–64). American Council on Education.

[CR16] Katsufrakis PJ, Chaudhry HJ (2021). Evolution of clinical skills assessment in the USMLE: Looking to the future after step 2 CS discontinuation. Academic Medicine.

[CR17] Kunutsor SK, Metcalf EP, Westacott R, Revell L, Blythe A (2022). Are remote clinical assessments a feasible and acceptable method of assessment? A systematic review. Medical Teacher.

[CR18] Langenau E, Kachur E, Horber D (2014). Web-based objective structured clinical examination with remote standardized patients and Skype: Resident experience. Patient Education and Counseling.

[CR20] Malau-Aduli BS, Jones K, Saad S, Richmond C (2022). Has the OSCE met its final demise? Rebalancing clinical assessment approaches in the peri-pandemic world. Frontiers in Medicine.

[CR21] Minty I, Lawson J, Guha P, Luo X, Malik R, Cerneviciute R, Kinross J, Martin G (2022). The use of mixed reality technology for the objective assessment of clinical skills: A validation study. BMC Medical Education.

[CR22] Monteiro S, Sullivan GM, Chan TM (2019). Generalizability theory made simple(r): An introductory primer to G-studies. Journal of Graduate Medical Education.

[CR23] Moss PA (1994). Can there be validity without reliability?. Educational Research.

[CR24] Motkur V, Bharadwaj A, Yogarajah N (2022). Is online objective structured clinical examination teaching an acceptable replacement in post-COVID-10 medical education in the United Kingdom? A descriptive study. Journal of Educational Evaluation for Health Professions.

[CR25] Oliven A, Nave R, Barch A (2011). Implementation of a web-based interactive virtual patient case simulation as a training and assessment tool for medical students. Studies in Health Technology and Informatics.

[CR26] Oliven A, Nave R, Baruch A (2021). Long experience with a web-based, interactive, conversational virtual patient case simulation for medical students’ evaluation: Comparison with oral ecamination. Medical Education Online.

[CR27] Papadakis MA (2004). The step 2 clinical-skills examination. New England Journal of Medicine.

[CR28] Park YS, Hyderi A, Bordage G, Xing K, Yudknowsky R (2016). Inter-rater reliability and generalizability of patient note scores using a scoring rubric based on the USMLE Step-2 CS format. Advances in Health Science Education.

[CR29] Patrício MF, Julião M, Fareleira F, Carneiro AV (2013). Is the OSCE a feasible tool to assess competencies in undergraduate medical education?. Medical Teacher.

[CR30] Porto FR, Ribeiro MA, Ferreira LA, Oliveira RG, Devito KL (2023). In-person and virtual assessment of oral radiology skills and competences by the Objective Structured Clinical Examination. Journal of Dental Education.

[CR31] Rentschler DD, Eaton J, Cappiello J, Mcnally SF, Mcwilliam P (2007). Evaluation of undergraduate students using objective structured clinical evaluation. Journal of Nursing Education.

[CR32] Saad SL, Richmond C, Jones K, Schilipalius M, Rienits H, Malau-Aduli BS (2022). Virtual OSCE Delivery and Quality Assurance during a pandemic: Implications for the future. Frontiers in Medicine.

[CR34] Shaban S, Tariq I, Elzubeir M, Alsuwaidi AR, Basheer A, Magzoub M (2021). Conducting online OSCEs aided by a novel time management web-based system. BMC Medical Education.

[CR35] Shaiba LA, Alnamnakani MA, Temsah M, Alamro N, Alsohime F, Alrabiaah A, Alanazi SN, Alhasan K, Alherbish A, Mobaireek KF, Bashiri FA, AlRuthia Y (2021). Medical Faculty’s and students’ perceptions toward Pediatric Electronic OSCE during the COVID-19 pandemic in Saudi Arabia. Healthcare.

[CR36] Shorbagi S, Sulaiman N, Hasswan A, Kaouas M, Al-Dijani MM, El-Hussein RA, Daghistani MT, Nugud S, Guraya SY (2022). Assessing the utility and efficacy of e-OSCE among undergraduate medical students during the COVID-19 pandemic. BMC Medical Education.

[CR33] Skrzypek N, Baster N, Perera I, Żądło A, Stalmach-Przygoda A, Szeliga M, Cebula G (2022). Adapting the OSCE to the times of the COVID-19 pandemic. A look at the e-OSCE from the students’ perspective. Folia Medica Cracoviensia.

[CR37] Streiner, D. L., Norman, G. R., & Cairney, J. (2015). *Health measurement scales: A practical guide to their development and use* (5th ed.). Oxford University Press.

[CR38] Tavakol M, Brennan RL (2013). Medical education assessment: A brief overview of concepts in generalizability theory. Int J Medical Education.

[CR39] Thampy, H., Collins, S., Baishnab, E., Grundy, J., Wilson, K., & Cappelli, T. (2022). Virtual clinical assessment in medical education: An investigation of online conference technology. *Journal of Computing in Higher Education*, 1–22. 10.1007/s12528-022-09313-6. 10.1007/s12528-022-09313-6 PMC9022162 35469333

[CR41] Trejo-Mejía JA, Sánchez-Mendiola M, Méndez-Ramírez I, Martínez-González A (2016). Reliability analysis of the objective structured clinical examination using generalizability theory. Medical Education Online.

[CR40] Tsichlis JT, Re D, Carmody JB (2021). The past, present, and future of the United States Medical Licensing Examination Step 2 clinical skills examination. Cureus.

[CR42] Vallevand A, Violato C (2012). A predictive and construct validity study of a high-stakes objective clinical examination for assessing the clinical competence of international medical graduates. Teaching and Learning in Medicine.

[CR43] Van der Vleuten, C. P. M., & Cohen-Schotanus, J. (2009). Standard setting. In N. Patil, & L. K. Chan (Eds.), *Assessment in medical and health sciences education* (pp. 62–71). Li Ka Shing Faculty of Medicine.

[CR44] Watkins MW (2018). Explratory factor analysis: A guide to best practive. Journal of Black Psychology.

